# Effectiveness of individual and group interventions for people with type 2 diabetes^1^

**DOI:** 10.1590/0104-1169.0247.2543

**Published:** 2015

**Authors:** Maria Fernanda Manoel Imazu, Barbara Nascimento Faria, Guilherme Oliveira de Arruda, Catarina Aparecida Sales, Sonia Silva Marcon

**Affiliations:** 2 MSc, RN, Confederação Nacional das Cooperativas Médicas - Unimed do Brasil, Londrina, PR, Brazil; 3 Statistician; 4 Doctoral student, Universidade Estadual de Maringá, Maringá, PR, Brazil. Scholarship holder from Coordenação de Aperfeiçoamento de Pessoal de Nível Superior (CAPES), Brazil; 5 PhD, Professor, Centro de Ciências da Saúde, Universidade Estadual de Maringá, Maringá, PR, Brazil

**Keywords:** Diabetes Mellitus, Health Education, Health Promotion, Nursing

## Abstract

**OBJECTIVE:**

to compare the effectiveness of two educational interventions used by a healthcare provider in the monitoring of individuals with type 2 diabetes mellitus (T2DM), regarding knowledge of the disease, impact on quality of life and adoption of self-care actions.

**METHODS:**

comparative, longitudinal, prospective study performed with 150 subjects with type 2 diabetes, analyzed according to the type of participation in the program (individual and/or group). Participants of the individual intervention (II) received nursing consultations every six months and those of the group intervention (GI) took part in weekly meetings for three months. Data were collected through four questionnaires: Identification questionnaire, Problem Areas in Diabetes Questionnaire (PAID), Summary of Diabetes Self-Care Activities Questionnaire (SDSCA) and the Diabetes Knowledge Scale (DKN-A). Data were analyzed using the Friedman and Mann Whitney tests, considering a statistical significance of p ≤ 0.05.

**RESULTS:**

there was an increase in knowledge about the disease in the II (p<0.003) and GI (p<0.007), with reduction of the impact on the quality of life in the II (p<0.007) and improvement in self-care actions in the GI (p<0.001).

**CONCLUSION:**

in both intervention models improvements were observed in the indicators, over the six month monitoring period.

## Introduction

The high rates of morbidity and mortality from type 2 diabetes mellitus (T2DM) determine the need for proposals for the reorientation of a healthcare model that prioritizes the practices that promote health and the integrality of the care^(^ 1 ^)^, in both the public and private sectors. Accordingly, the Ministry of Health, through the National Health Agency (ANS), stimulated changes in private healthcare providers, and the Normative Resolution (RN) No. 94, of 2005, established the criteria for the development of health promotion programs^(^ 2 ^)^. This is due to health promotion being a community empowerment process in the practice of improving the quality of life and health, including greater participation in the control of this process, and contributing to the development of integral healthcare^(^ 3 ^)^.

In this context, health education is the theoretical and methodological basis for health promotion actions, as it can support both diseases prevention and rehabilitation and promote citizenship, personal and social responsibility related to health and contribute in the training of multipliers and caregivers^(^ 4 ^)^. Thus, health promotion and health education are strictly linked, considering that for effective health promotion it is necessary to articulate technical and popular knowledge, and mobilize institutional and community, public and private resources. Thus, health education constitutes a tool to improve individual and collective health conditions, reinforcing the maintenance of positive health habits through a multi-dimensional approach toward the health-disease process^(^ 5 ^)^.

Health education is now considered a social process, which is defined as any influence experienced by individuals, capable of modifying their behavior. It is related to the implementation of problem-solving activities by health professionals, which valorize the everyday experience of individuals and social groups, and encourage the active participation of the learner in the educational process. It involves the adoption of approaches systematically planned and implemented in a non-coercive manner^(^ 5 ^)^. Thus, health education differs from the traditional model of knowledge transmission.

Accordingly, educational activities, in which a key element is health education, are experiences materialized in organized and systematized activities, inherent to the healthcare project at all levels of care. They permit the appropriation of knowledge, improvement of the quality of life of the population, reduction of problems and damage originating from the diseases and a critical reflection regarding the actions necessary to resolve such problems, involving system users and health professionals, especially nurses^(^ 6 ^)^.

Therefore, for educative actions to generate learning, it is necessary for them to be based on an accessible and emancipatory type of health education, that is, the dialogic model of health education that is primed by problematization, the construction of knowledge and skills, and based on dialogue, prolonged changes in behavior and greater autonomy for the individual^(^ 7 ^)^.

In this sense, the national and international literature on health education and T2DM, produced between 1997 and 2007, shows that the majority of studies were experimental and employed the following strategies: interactive education, community educational intervention, operative groups, seminars, monitoring of clinical and biochemical parameters, home visits, educational conferences, activities regarding nutrition and physical exercise, ophthalmological exams, case reports and educational colonies^(^ 8 ^)^.

It should be noted that studies on educational activities based on dialogic health education and developed in the private sector with individuals with T2DM are still incipient. Despite this gap in the literature, a study that aimed to analyze health promotion actions in diabetes education, developed in groups of a private healthcare provider, reports that the activities were dynamic and driven by the needs cited by the participants. In the same study, the authors consider the importance of studies that aim to evaluate the effectiveness of the educational programs, aiming to support the redirection of new strategies also within the private health context^(^ 9 ^)^. Therefore, among the relevant factors to be considered in the evaluation of such programs, the literature highlights knowledge about the disease, the impact of diabetes on the quality of life and the adoption of self-care actions, which may predict disease control in the daily life of the individual^(^ 10 ^-^ 12 ^)^.

In accordance with the above, in a study that evaluated the effectiveness of individual and group intervention offered by the outpatient clinic of a public hospital in Belo Horizonte, the authors found that the results of both strategies were similar regarding attitudes, changes of behavior and quality of life, however, with greater effectiveness in the group intervention, with regards to laboratory exams^(^ 9 ^)^.

These findings highlight the importance of knowing the effectiveness of different types of educative health actions, in the context of a private healthcare provider. Thus, the following research question arose: is there a significant difference between individual and group educational interventions, related to the effectiveness regarding knowledge about the disease, the impact of diabetes on the quality of life and the adoption of self-care actions? Linked to the research question, the aim of this study was to compare the effectiveness of two educational interventions used by a healthcare provider in the monitoring of individuals with type 2 diabetes mellitus (T2DM), regarding knowledge of the disease, impact on quality of life and adoption of self-care actions.

Therefore, aiming for collaboration to overcome the traditional model of health education, the present study proposed the implementation of individual and collective educational activities, mainly based on dialogue, with individuals with T2DM.

## Methods

This was a comparative, longitudinal and prospective study, developed in a healthcare service of Londrina, state of Paraná. The choice of this institution was made due it offering a "Chronic Patient Monitoring Program", which aims to perform health education, and more specifically, to encourage self-care, behavior change, improved quality of life and the reduction of healthcare costs.

People with hypertension and diabetes are included in the program and monitored by private physicians. The program consists of biannual nursing consultations, telephone monitoring and educational group activities. During the nursing consultation a physical examination, anamnesis and general guidance regarding the disease, treatment and self-care attitudes are carried out. Telephone monitoring is performed by the same nurse responsible for accompanying the patient, three months after the consultations. During this monitoring a pre-established script is used to identify attitudes that favor self-care, with an important focus on the use of medicines, doubts and problems that arise in the daily life. The group health education activities are provided for people with available time and interest. The group educational intervention consisted of 12 weekly meetings, with duration of 120 minutes each, for a period of three months, being held on fixed dates and times. The groups are conducted by a multidisciplinary team, consisting of a nurse, nutritionist, psychologist and social worker.

In the groups, health education activities are carried out in order to encourage changes in habits related to nutrition and disease care at the family and individual levels. The expectations and doubts of the participants regarding a particular theme are identified at each meeting. Entertainment and audiovisual resources - figures, fictional food and posters, are used in the discussion of these aspects.

For the present study, a population was selected by convenience from a specific field of study: individuals who were enrolled in the Monitoring Program, between October 2011 and February 2012. All individuals with T2DM (with or without comorbidities), of both genders, over 18 years of age, and enrolled in the program during this period were invited to participate in the study while waiting to be attended.

In the period mentioned above, of the 270 subjects enrolled, 85 did not have a diagnosis of T2DM, and of the 185 who met the inclusion criteria, 35 refused to participate in the study. Therefore, 150 subjects with T2DM were effectively studied, who, for the purposes of the study, were divided between individual and group interventions, according to option/availability, as the health service provider, being private, did not allow the participants to be allocated randomly.

Data were collected through semi-structured interviews, conducted in a private room within the institution, between October 2011 and July 2012, at three different moments, according to the trajectory of the patients in the service: the first moment (M1) occurred at the time of inclusion into the program, during the first nursing consultation; the second moment (M2) occurred three months later, during the telephone contact; and the third moment (M3), six months after the first, during the second nursing consultation. It should be noted that, as a function of this investigation, guidance and clarification of doubts, usually performed during the consultations and telephone contacts, throughout the study period, only occurred after the application of the data collection instruments. In total, four questionnaires were used, as described below.

- Identification questionnaire - applied only at the first moment, consisting of open and closed questions that addressed: a) sociodemographic characteristics (gender, age, race, marital status, education and individual income according to the minimum wage) and b) clinical characteristics: presence of comorbidity (hypertension, dyslipidemia, obesity, others); time of diagnosis of diabetes; use of diabetes medications; and data regarding the clinical and laboratory examinations. Data for glycated hemoglobin were obtained from consultation of patient records and capillary blood glucose was verified at the three moments. Controlled postprandial glycemia was considered when ≤ 160 mg/dl and uncontrolled glycemia when > 160 mg/dl, good glycemic control of glycated hemoglobin was considered when ≤ 7%, and inadequate glycemic control when > 7% mg/dl^(^ 13 ^)^.

- Problem Areas in Diabetes Questionnaire (PAID), validated in Brazil^(^ 14 ^)^, consisting of 20 questions, distributed over four dimensions: emotional, food-related, social support and treatment problems. The total score ranges from 0-100 points, with higher scores indicating high levels of emotional distress^(^ 14 ^)^.

- Summary of Diabetes Self-Care Activities Questionnaire (SDSCA), validated in Brazil^(^ 15 ^)^, consisting of 17 items, distributed over six dimensions, which enables the evaluation of adherence to self-care activities, taking as reference the frequency with which certain activities were carried out in the previous seven days. In the analysis of the adherence, the questionnaire items were parameterized in number of days of the week, from zero to seven, zero being the worst possible situation and seven the more favorable. In the items that assess the consumption of foods high in fat and sugar, the values are reversed^(^ 15 ^)^.

- Diabetes Knowledge Scale (DKN-A), also validated in Brazil^(^ 16 ^)^, consisting of 15 items related to the general knowledge of DM, which involves: basic physiology, food groups and their replacements, DM management in situations of complications, and general principles of disease care^(^ 16 ^)^. The responses are presented in a multiple choice scale and the total score ranges from zero to 15 points, with scores lower than seven indicating unsatisfactory knowledge, and scores equal to or greater than eight indicating satisfactory knowledge^(^ 10 ^)^.

The data from individuals who participated in at least eight of the 12 meetings held were considered for analysis. The IBM SPSS 20. software was used for the performance of the statistical tests To verify that the groups of participants were comparable in terms of sociodemographic and clinical variables, at the moment before the interventions, the nonparametric test of proportions was applied. The only significant difference was in the use of antidiabetic medication, thus demonstrating that the groups were not statistically different in the majority of the variables used, enabling comparisons to be performed.

Based on the Kolmogorov-Smirnov and Shapiro-Wilk tests, the data distribution was considered non-normal. Therefore, Friedman's test was used for the comparison of each type of intervention, at the three different observation moments, and Friedman's Multiple Comparison test was performed to verify the difference, to evidence at exactly which moments the differences occurred; and the Mann Whitney test to compare the results of the Individual Intervention with those of the Group Intervention, at each moment. In all tests, the level of significance was set as p-value ≤ 0.05.

The development of the study met the national and international standards of ethics in research involving human subjects, according to Resolution 196/96 of the National Health Council. The project that gave rise to this study was approved by the Permanent Committee of Ethics in Research with Human Subjects (COPEP), of the State University of Maringá (Authorization No. 516/2011). All participants, after clarification of the objectives and criteria for participation, signed two copies of the Informed Consent Form (ICF).

## Results

Of the 150 individuals included in the study, 120 (80%) participated in the second assessment, and 114 (76%) in the third. Considering the mode of intervention, the loss of 31 subjects (28.9%) was verified from the individual intervention and five (11.63%) from the group intervention. Among the reasons for leaving, 28 cases were due to termination of the health plan, six due to change of city and two due to serious complications in the health status.

Regarding the initial assessment, it was found that the 150 individuals participating in the study had a mean age of 60 years (± 12.49 years), a mean individual of income of 5.5 minimum wages (± 9.85), more than half (56%) were female, and the majority were white ( 80%), lived with a partner (74%), and had more than eight years of education (64%). It was also found that the majority of the participants had satisfactory knowledge about the disease (71.3%), perceived a high impact on their quality of life (76%) and presented good adherence to self-care practices.

Table 1 shows that there was a significant increase in the median values obtained regarding knowledge, for both types of intervention; and that there was an increase regarding self-care only in the group intervention, while a significant reduction of the impact of the disease on the quality of life only occurred in the individual intervention. More specifically, there was a significant increase in the level of knowledge about the disease from M1 to M3, in the two types of intervention. Regarding the impact of the disease on the quality of life, there was a significant reduction of the scores from M1 to M2 and from M1 to M3 only among the individual intervention participants. Finally, there was a significant increase in the self-care median scores from M1 to M2 and from M1 to M3, only among the group intervention participants.

**Table 1 -:** Distribution of knowledge, impact of disease and self-care median scores in patients with diabetes enrolled with a healthcare service provider, at the three moments of individual and group interventions. Londrina, PR, Brazil, 2012

Variables^||^	INDIVIDUAL				GROUP			
	M1^*^	M2^†^	M3^‡^	p^§^	M1	M2	M3	p
	n=107	n=82	n=76		n=43	n=38	n=38	
Knowledge about diabetes (DKN-A)	9.0	10.0	10.0^¶^	0.003	9.0	10.0	11^¶^	0.008
Impact of disease on QoL (PAID)	28.0	16.0^¶^	15.0^¶^	0.007	26.0	18.0	13.0	0.140
Self-care (SDSCA)	3.0	4.0	4.0	0.085	3.0	4.0^¶^	4.0^¶^	<0.001

* M1: Moment 1 (start);

† M2: Moment 2 (three months of intervention);

‡ M3: Moment 3 (six months of intervention);

§ Friedman's Test for three paired groups;

|| The median values were rounded up.

¶ Friedman's Multiple Comparisons: different from M1.

It can be observed in Table 2, when comparing the two groups, at the different moments, that no statistically significant differences were found for any of the variables under study.

**Table 2 -:** Comparison of scores on the knowledge on diabetes, impact on quality of life and self-care, obtained at the three moments, according to the types of intervention. Londrina, PR, Brazil, 2012

Variables^||^	M1^*^		p^§^	M2^†^		p	M3^‡^		p
	(n = 150)			(n = 120)			(n = 114)		
	Individual	Group		Individual	Group		Individual	Group	
Knowledge about diabetes (DKN-A)	9.0	9.0	0.410	10.0	10.0	0.815	10.0	11.0	0.894
Impact of disease on QoL (PAID)	28.0	26.0	0.227	16.0	18.0	0.703	15.0	13.0	0.597
Self-care (SDSCA)	3.0	3.0	0.539	4.0	4.0	0.935	4.0	4.0	0.967

* M1: Moment 1 (start);

† M2: Moment 2 (three months of intervention);

‡ M3: Moment 3 (six months of intervention);

§ Mann Whitney Nonparametric Test.

|| The median values were rounded up.

## Discussion

The data from this study show improvements in the scores related to knowledge about the disease, after six months of intervention, in both groups; improvements in the scores related to the impact of the disease on the quality of life, after three and six months, only for the individual intervention; and a positive influence regarding the adherence to self-care practices, at three and six months, only for the group intervention.

Thus, these findings corroborate the results of an experimental study^(^ 9 ^)^ conducted with T2DM subjects, which also identified the effectiveness of both types of intervention, individual and group, at different moments and referred to the same aspects evaluated. However, in the present study, the group intervention constituted an extra activity in relation to the individual intervention, in that its participants took part in both approaches. Educational activities implemented by health professionals together with individuals, families and community, are essential for controlling this disease, as the complications of diabetes are directly related to knowledge about the disease, considering that this supports the performance of daily self-care and the adoption of a healthier lifestyle^(^ 17 ^)^. Individual or group interventions comprise the educational strategies most commonly used in the health promotion and monitoring of patients with diabetes^(^ 18 ^)^. However, it is important to consider that certain educational strategies can encourage the active participation of the individual in controlling the disease and preventing its complications or, on the contrary, simply strengthen the curative character focused on the disease and on the transmission of information^(^ 19 ^)^.

The number of individuals who, at the beginning of the program, already presented satisfactory knowledge regarding the disease was high, a fact that differs from a similar study conducted in a primary health unit, in which the level of knowledge was considered unsatisfactory for the majority of the individuals, which was shown to be associated with the low educational level of the study participants^(^ 12 ^)^. In addition, in the present study, an increase of values related to knowledge of the disease was observed over six months, both among participants of the individual intervention as well as among those who participated in the group intervention. Strategies aimed at health education should be employed, aiming for the development of self-care^(^ 13 ^)^. In health education, one of the indicators most used for assessment in diabetic patients has been the level of knowledge about the disease, as this variable is related to the efficacy of the program^(^ 20 ^)^.

Regarding the impact of the disease on the quality of life, it was observed that at the start of the intervention individuals perceived a high impact of diabetes in their lives, and after six months there was a reduction of this impact, being significant for the participants of the individual intervention. These data show how the performance of monitoring of these individuals can benefit them, as highlighted by the literature, which refers to the care of the disease, coping with emotional imbalances, management of the treatment and improved quality of life^(^ 21 ^)^, therefore the presence of the diagnosis of diabetes influences the self-perception of physical and psychological well-being^(^ 22 ^)^.

A study conducted in Denmark, with 143 T2DM individuals, found no significant difference between individual and group interventions with regard to improving the quality of life, except for the improvement of clinical data in the individual intervention, which may have contributed to these individuals evidencing a lower impact of the disease^(^ 23 ^)^. Therefore, in line with the results of the present study, it has been concluded that individual monitoring by the nurse, directed toward self-management and disease control, helps to reduce the impact on the quality of life of individuals^(^ 24 ^)^_._

However, it should be emphasized that statistically significant decreases in the impact of the disease on the quality of life occurred in the individuals who only participated in the nursing consultations (individual intervention). This may be related, in part, to the small number of group intervention participants, undermining the identification of associations even when they existed.

In clinical terms, the absence of changes in the impact of the disease among the group intervention participants could be, on one hand, due to a possible lack of motivation of some people, when they realized that other participants improved their quality of life, causing them to become despondent, due to their difficulties. On the other hand, the possibility of nurses not valuing the moment of individual care should be considered, as they believed that the patients were being well assisted in the group and, therefore, gave more time and care to those that only received the nursing consultation.

In relation to self-care, an increase of positive actions (improvement in eating habits and the practice of physical exercise) was identified in participants of both groups. However, after six months, this reduction was only significant for the individuals that also participated in the group intervention. This data can be related to the fact that the group activities make possible dialogue, reflection, exchange of knowledge and, consequently, the co-responsibility of individuals with diabetes for their own health. In addition, the participation of a multidisciplinary team in the group activity enables the integrality of the healthcare, as it favors the contact with and access to knowledge of different health professionals^(^ 24 ^)^. The multidisciplinary team, therefore, tends to favor the reduction of the stress associated with the disease, receptivity to the treatment, self-esteem, sense of self-efficacy and a more positive perception regarding the health^(^ 10 ^)^.

Furthermore, the commitment of individuals to self-care practices largely depends on cultural and educational aspects related to personal skills and limitations, life experience, health status and the resources available. When an individual is unable to meet their self-care requirements, it is the function of the nurse and healthcare care team to determine at what level this occurs, defining the necessary methods of support^(^ 25 ^)^.

Finally, the allocation of the participants into groups by convenience, no control of variables e.g. the presence of comorbidities, and also the rate of loss in the groups were considered limitations. Regarding the group intervention, it is emphasized that the non-randomization of the participants resulted in the inclusion of people who had already shown an interest in participating in this activity, which may have facilitated the change in self-care attitudes. There was also a large difference between the educational approaches, in terms of the frequency of meetings, with the members of the individual intervention participating in only the two nursing consultations (semiannual) and the telephone contact, while those of the group intervention participated in these same activities and at least eight weekly meetings.

Despite these limitations, the results are valid, especially when comparing the behavior of the groups at the three different moments. They can support and motivate the professional practice in the implementation of health education activities in different contexts.

## Conclusion

The study results show that the individual intervention for people with T2DM, through biannual nursing consultations, favored the clarification of doubts, the acquisition of knowledge about the disease and the reduction of its impact on the quality of life of the individuals. Furthermore, by adding health education actions in groups to these consultations, in addition to promoting the acquisition of knowledge, the occurrence of greater adherence to self-care practices was also observed, although the reduction of the impact of the disease on the quality of life of patients was not significant.

The results also showed that in the two types of intervention significant changes occurred from M1 to M2 or M3, but not from M2 to M3. This means, on one hand, that the acquired knowledge and behavior have permanence in time, as they did not decrease from M1 to M3; while on the other hand, apart from the influence of personal motivation, usually observed at the beginning of the interventions, it was found that people have a limit for the acquisition of knowledge and behavioral skills. These particularities indicate that health professionals who treat patients with chronic diseases, such as T2DM, must consider the need for adjustments in the educational program, aiming to ensure the maintenance of the benefits achieved, regardless of the type of educational intervention used.

Thus, the performance of studies to assess the effects of the interventions 12 months after their completion is proposed, in order to identify whether the benefits remain over the long term, which would characterize the residual effect of interventions previously performed. Therefore, it is appropriate to propose the continuation of studies of this nature, through qualitative methodologies that enable the identification of the elements that contribute to changes, the comprehension of how these elements work in these changes, and the clarification of the boundaries between individual and group interventions in the modulation of self-care, considering that the educational process constitutes something dynamic and therefore subject to continuous and multidimensional assessment.
